# Effects of geographical, soil and climatic factors on the two marker secondary metabolites contents in the roots of *Rubia cordifolia* L.

**DOI:** 10.3389/fpls.2024.1419392

**Published:** 2024-06-11

**Authors:** Yanlin Wang, Huanchu Liu, Shuai Yu, Yue Zhang, Yanqing Huang, Xingyuan He, Wei Chen

**Affiliations:** ^1^ CAS Key Laboratory of Forest Ecology and Silviculture, Institute of Applied Ecology, Chinese Academy of Sciences, Shenyang, China; ^2^ University of Chinese Academy of Sciences, Beijing, China; ^3^ Liaoning Shenyang Urban Ecosystem National Observation and Research Station, Shenyang, China; ^4^ Shenyang Arboretum, Chinese Academy of Sciences, Shenyang, China

**Keywords:** *Rubia cordifolia* L., purpurin, mollugin, environmental factors, planting

## Abstract

The growth and quality of medicinal plants depend heavily on environmental variables. The quality of *Rubia cordifolia*, an important medicinal plant, is determined by the two main secondary metabolites of the root, purpurin and mollugin. However, their relationship with environmental factors has not been studied. In this study, the purpurin and mollugin contents of *R. cordifolia* roots from different sampling sites in China were measured using ultra-high-performance liquid chromatography, and the correlations between the two secondary metabolites and environmental variables were analyzed. The results showed that there were significant differences in the contents of purpurin and mollugin in the roots of *R. cordifolia* at different sampling points. The content of purpurin ranged from 0.00 to 3.03 mg g^-1^, while the content of mollugin ranged from 0.03 to 10.09 mg g^-1^. The quality of *R. cordifolia* in Shanxi, Shaanxi and Henan border areas and southeastern Liaoning was higher. Liaoning is expected to become a *R. cordifolia* planting area in Northeast China. Correlation and regression analysis revealed that the two secondary metabolites were affected by different environmental factors, the two secondary metabolites contents were positively correlated with longitude and latitude, and negatively correlated with soil nutrients. In addition, higher temperature and shorter sunshine duration facilitated the synthesis of purpurin. Annual precipitation might be the main factor limiting the quality of *R. cordifolia* because it had opposite effects on the synthesis of two major secondary metabolites. Therefore, this study is of great significance for the selection of *R. cordifolia* planting areas and the improvement of field planting quality.

## 1 Introduction

Traditional Chinese Medicine (TCM) has been extensively used to treat various illnesses in China for thousands of years (Wang et al., 2021a). TCM continues to play a key role in safeguarding the health of Chinese people and is attracting increasing global attention (Xue et al., 2012). The idea of integrating Chinese and Western medicines to treat complex disorders has also been adopted by the modern medical community, underscoring the significance of TCM. Since the outbreak of the novel coronavirus pneumonia in 2019, TCM has demonstrated significant promise for the management of COVID-19 (Du et al., 2020; Lyu et al., 2021; Zhao et al., 2021).

The continual development of TCM requires support for further studies on medicinal plants. Only high-quality medicinal plants can be used to treat various diseases. The quality of medicinal plants is not only related to the clinical effect of Chinese medicine but also plays a key role in developing the Chinese medicine industry (Qin et al., 2018). The active components of Chinese medicine are the basis for the efficacy of Chinese medicine, and these innovative drugs are mainly extracted from medicinal plants (Ma et al., 2022). For example, the well-known antimalarial drug artemisinin is extracted from the medicinal plant *Artemisia annua* L (Tu, 1999).

**Figure 1:**
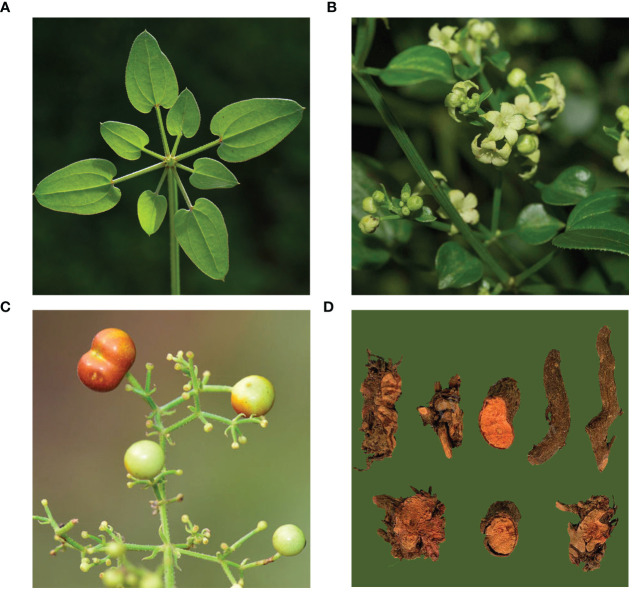
Photographs of *R. cordifolia*. **(A)** Leaf; **(B)** Flower; **(C)** Fruit; **(D)** Root. **(A–C)** were obtained from iNaturalist (https://www.inaturalist.org/), and **(D)** was obtained from iPlant (http://www.iplant.cn/).

*Rubia cordifolia* L. is a medicinal plant whose roots can be used to treat various diseases owing to its anti-inflammatory, anticancer, and anti-platelet aggregation properties as various secondary metabolites, including anthraquinones and naphthoquinones, are present in its roots (**Figure 1**) (Wen et al., 2022). The Chinese Pharmacopoeia usually specifies standards for Chinese herbal medicines, which stipulate that the two main compounds in the roots of *R. cordifolia*, that is, the purpurin content is not less than 0.1% and the mollugin content is not less than 0.4% (Chinese Pharmacopeia Commission, 2020). Purpurin has anticancer, antibacterial, and neuromodulatory effects owing to its strong antioxidant activity (Singh et al., 2021). Similarly, mollugin has a variety of pharmacological effects, including neuroprotective, anti-inflammatory, anticancer, and antiviral effects (Kim et al., 2013; Wang et al., 2017; Li et al., 2020). Therefore, the contents of both compounds are essential for the quality of *R. cordifolia*.

Plant secondary metabolites are not only important for humans but also vital for themselves. Plants synthesize and accumulate various secondary metabolites to resist possible stresses caused by the external environment (Selmar and Kleinwächter, 2013; Loreto et al., 2014). For example, psoralen, the main secondary metabolite in *Psoralea corylifolia* L., increases under chromium and UV-B stress (Pandey et al., 2023). By contrast, a short drought stress period increased the amount of saponins in the roots of *Bupleurum chinense* DC (Yang et al., 2019a). However, an unfavorable growth environment may contribute to the synthesis and accumulation of secondary metabolites but may not be conducive to plant growth, resulting in reduced yield. Further research has shown that medicinal plants grow more effectively and to a higher quality in their appropriate habitats, which is strongly related to the environment in the habitat (Zhan et al., 2022; Zheng et al., 2022). The synthesis and accumulation of secondary metabolites in medicinal plants are affected by a combination of factors.

Although *R. cordifolia* has long been used as a medicinal plant, research on *R. cordifolia* has mainly focused on the discovery of its secondary metabolites and associated pharmacological effects (Shan et al., 2016; Wen et al., 2022). The quality and productivity of medicinal plants can be improved by understanding the relationship between environmental conditions and secondary metabolite content. Environmental factors may also influence the content of secondary metabolites in *R. cordifolia*; however, little work has been conducted on *R. cordifolia* in this area. Therefore, the objectives of this study were to (1) collect *R. cordifolia* throughout China and determine the content of two major secondary metabolites in the roots at each location; (2) analyze the environmental conditions of the sampling sites, including soil, climate, and topography; and (3) analyze the relationship between secondary metabolite content and environmental factors at each location to explore the primary environmental variables affecting the synthesis and accumulation of *R. cordifolia* root secondary metabolites.

## 2 Materials and methods

### 2.1 Sampling

**Figure 2:**
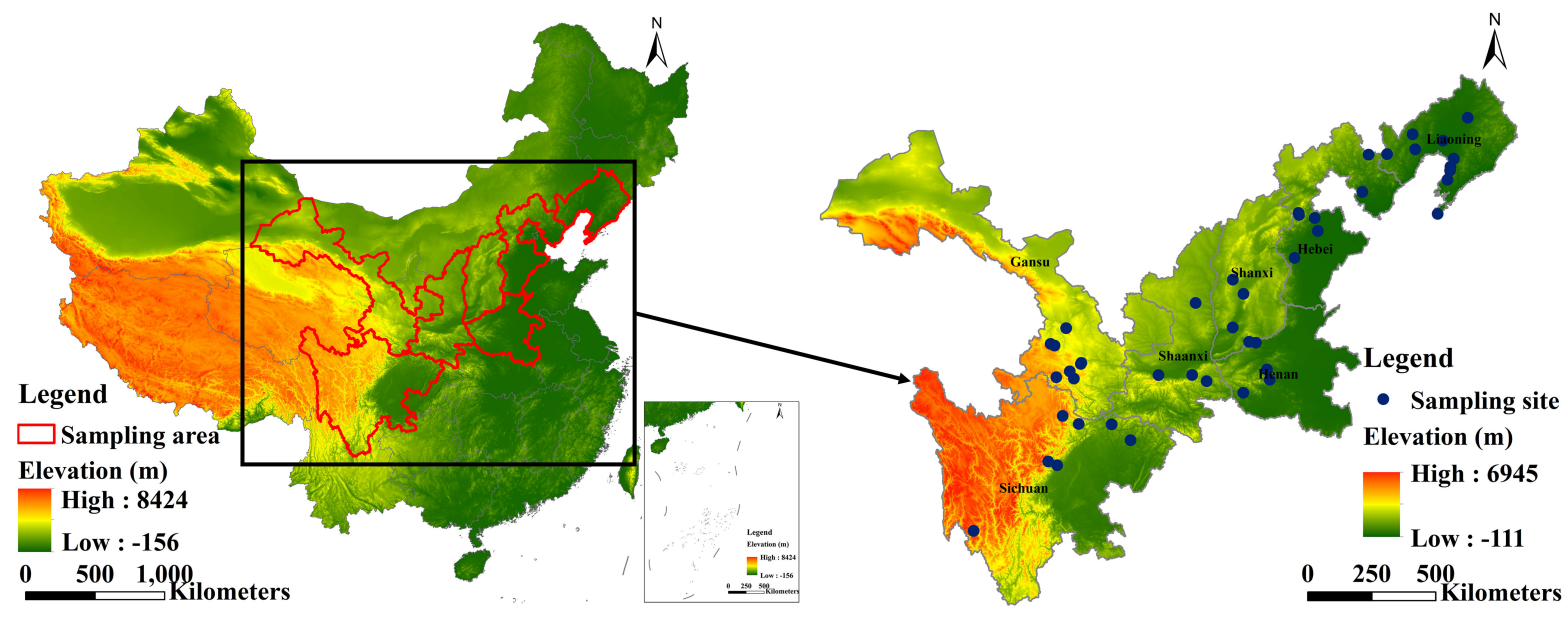
Study area and distribution of sampling sites.

Based on the distribution of *R. cordifolia* and its origin, as described in the literature (Xue et al., 2009; Yu et al., 2017; Yan, 2021), Liaoning, Hebei, Henan, Shanxi, Shaanxi, Gansu, and Sichuan were selected for our sampling (**Figure 2**). Samples were collected from July to August in 2021 and 2022, and 3–7 whole underground parts of *R. cordifolia* were collected from each sample point, while trying to ensure that the samples had the same plant age. Soil at 0–30 cm depth in the roots was collected for subsequent analysis. The coordinates of each sampling point were recorded for the subsequent acquisition of meteorological and topographical factors, as shown in **Table 1**.

**Table 1:** Sampling sites information of *R. cordifolia*.

Sampling site	Province	Long (°E)	Lat (°N)	Ele (m)	pH	OM (g kg^-1^)	AN (mg kg^-1^)	AP (mg kg^-1^)	AK (mg kg^-1^)	TEM (°C)	SSD (h)	PRE (mm)
S1	Gansu	104.568	34.615	2314	8.22	26.87	99.68	9.20	99.28	550.06	2063.25	7.60
S2	Gansu	104.591	34.650	1773	8.53	22.42	98.01	10.25	262.23	470.75	2043.05	9.46
S3	Gansu	103.909	35.893	2420	7.91	65.60	284.41	40.70	686.08	542.15	2459.26	4.59
S4	Gansu	103.909	35.893	2420	7.93	156.35	557.04	13.17	263.13	542.15	2459.26	4.59
S5	Gansu	104.089	34.342	2453	8.22	46.06	192.74	17.30	504.34	658.68	2066.46	6.47
S6	Gansu	104.275	34.085	2107	8.19	54.17	224.38	43.73	891.55	637.46	1936.29	8.70
S7	Gansu	103.411	35.256	2559	8.07	53.25	223.32	13.40	301.15	622.20	2324.24	5.06
S8	Gansu	103.238	35.317	2457	7.99	100.23	445.02	13.47	281.47	637.76	2341.98	4.81
S9	Gansu	103.517	34.124	3045	6.92	241.37	1121.45	14.30	357.81	742.23	2184.48	4.25
S10	Hebei	115.511	39.342	54	8.48	14.56	72.96	16.73	122.67	504.51	2216.87	12.58
S11	Hebei	117.909	40.019	69	7.26	24.99	132.61	14.00	223.80	624.89	2439.15	12.16
S12	Hebei	114.354	38.000	112	8.30	15.83	63.26	8.00	100.02	505.47	2162.80	14.02
S13	Hebei	118.504	41.324	1063	7.02	88.13	379.07	18.13	746.99	565.10	2690.20	5.11
S14	Hebei	114.799	39.528	1067	8.42	18.36	84.64	7.17	188.46	543.41	2509.13	7.46
S15	Hebei	114.788	39.606	1543	8.13	53.55	201.25	12.27	122.92	620.36	2578.69	4.96
S16	Hebei	115.579	38.862	14	8.33	29.97	140.56	100.23	382.16	490.43	2221.99	12.95
S17	Henan	112.601	34.112	377	7.33	28.02	120.52	15.76	205.64	657.62	1842.75	14.95
S18	Henan	111.505	33.356	324	7.80	26.18	109.05	30.87	71.76	795.80	1813.56	15.67
S19	Henan	112.674	33.729	193	6.64	60.28	266.27	33.25	300.64	749.14	1758.83	15.47
S20	Henan	112.233	35.103	496	8.13	28.47	96.22	24.63	252.01	657.78	2038.04	13.65
S21	Liaoning	122.490	40.569	18	7.99	22.16	96.19	21.10	161.43	677.13	2550.11	9.90
S22	Liaoning	122.186	40.181	73	6.45	25.89	118.76	15.00	179.02	667.86	2590.11	9.75
S23	Liaoning	121.972	39.873	69	7.34	22.31	129.30	36.17	518.81	655.27	2560.75	9.99
S24	Liaoning	122.239	40.310	80	6.63	38.95	205.31	73.18	223.42	678.32	2588.21	9.67
S25	Liaoning	123.589	41.908	110	6.13	29.24	150.83	18.60	160.69	690.18	2404.79	8.32
S26	Liaoning	122.169	41.299	5	7.71	10.02	41.18	23.97	136.25	629.54	2566.43	9.61
S27	Liaoning	119.391	41.221	436	8.27	34.91	139.81	37.50	337.35	484.38	2677.92	8.65
S28	Liaoning	121.192	38.734	165	6.31	67.86	304.80	12.77	496.08	643.76	2515.16	10.84
S29	Liaoning	120.770	41.199	93	7.73	92.56	164.82	23.33	517.95	505.58	2596.92	9.76
S30	Liaoning	120.786	41.756	144	7.88	87.06	340.89	53.10	306.84	446.45	2653.15	9.30
S31	Liaoning	120.786	41.756	144	8.26	19.56	88.24	12.23	337.17	446.45	2653.15	9.30
S32	Shanxi	111.942	35.153	481	8.36	24.31	86.57	31.08	360.97	640.74	2036.07	13.79
S33	Shanxi	111.456	37.438	1347	8.45	19.99	78.79	34.87	193.37	605.00	2412.83	8.13
S34	Shanxi	111.275	35.721	448	8.41	37.01	78.16	36.77	312.10	483.95	2091.55	14.21
S35	Shanxi	111.881	36.896	916	8.28	33.58	122.30	33.00	277.46	542.56	2197.33	10.79
S36	Shaanxi	109.705	36.695	947	8.49	12.19	41.38	14.15	102.70	558.48	2365.28	10.36
S37	Shaanxi	109.979	33.865	771	7.10	25.46	122.09	5.87	137.81	704.92	1918.56	13.24
S38	Shaanxi	107.919	34.163	544	7.88	44.24	160.73	21.37	189.28	687.90	1796.05	13.79
S39	Shaanxi	109.374	34.114	686	7.47	26.16	84.93	13.08	213.20	732.79	1933.74	12.87
S40	Sichuan	103.815	32.748	3395	7.09	334.23	1168.00	36.90	402.24	939.84	1815.98	3.12
S41	Sichuan	104.497	32.463	1012	8.19	54.43	211.21	11.80	175.53	873.64	1414.93	13.76
S42	Sichuan	105.880	32.447	540	8.54	16.05	60.32	6.10	112.61	1114.55	1315.61	16.10
S43	Sichuan	103.616	30.989	721	8.17	46.68	179.02	15.67	271.32	1067.28	1037.23	16.40
S44	Sichuan	100.349	28.566	3012	7.86	100.78	400.23	91.53	1038.73	505.66	2312.12	9.76
S45	Sichuan	106.660	31.870	436	8.38	26.07	170.23	7.23	96.28	1206.15	1323.29	17.31
S46	Sichuan	103.250	31.118	1901	8.15	31.71	108.10	11.83	75.15	1084.87	1304.95	8.38

Long, Longitude; Lat, Latitude; Ele, Elevation; OM, Organic matter; AN, Available nitrogen; AP, Available phosphorus; AK, Available potassium; TEM, Annual mean temperature; SSD, Annual sunshine hours; PRE, Annual precipitation.

### 2.2 Extraction and detection of secondary metabolites

The *R. cordifolia* root powder (0.5 g) was precisely weighed and placed in a stoppered conical flask. Methanol (100 mL) was added to the flask and stored it overnight. Secondary metabolites were extracted using ultrasound (power 250W, frequency 40kHz, 30 minutes). After the ultrasound was completed, the sample was cooled to room temperature, shaken well, and filtered. The filtrate (50 mL) was measured precisely, and evaporate to dryness in a 60°C water bath. The residue was dissolved by adding 20 mL methanol:25% hydrochloric acid (4:1). The solution was heated and hydrolyzed in an 80°C water bath for 30 minutes. The solution obtained after hydrolysis was cooled immediately, 3 ml of triethylamine was added, mixed, transferred to a 25 ml measuring flask, and diluted to volume with methanol. The solution was filtered through a microporous membrane and used for detection (Chinese Pharmacopeia Commission, 2020).

Ultra-high-performance liquid chromatography (UHPLC) (Ultimate 3000 RSLC, Thermo Fisher Scientific, MA, USA) was used to determine the mollugin and purpurin contents in the extract. Octadecylsilane bonded silica gel was used as the filler of chromatographic column, and the 25:50:25 mixture of methanol, acetonitrile, and 0.2% phosphoric acid was used as the mobile phase The detection wavelength is 250 nm. According to the calculations of mollugin and purpurin peaks, the theoretical plate number should not be less than 4000.

### 2.3 Soil sample processing and analysis

Soil samples were taken back to the laboratory and sieved to remove stones, fine roots, and other debris. The soil was naturally dried and ground, and part of the ground samples were passed through a 0.25 mm sieve to determine the organic matter content of the soil. The remaining samples were sieved through a 1 mm sieve, and the soil pH, available nitrogen, available phosphorus, and available potassium were determined using the methods described by Hu and Wang (2020). Soil pH was determined by the potentiometric method, organic matter content was determined by the potassium dichromate volumetric method, available nitrogen content was determined by the alkaline hydrolysis diffusion method, available phosphorus content was determined by the NaHCO_3_ extraction-colorimetric method, and available potassium content was determined by the ammonium acetate extraction-flame photometric method.

### 2.4 Access to climate and geographic factors

The annual mean temperature (TEM), annual precipitation (PRE), and annual sunshine hours (SSD) for 2010–2020 were obtained from the Resource and Environment Science and Data Center (https://www.resdc.cn/) (Xu, 2017). Elevation data were obtained from WorldClim (https://www.worldclim.org/). Finally, the climatic and geographic factors of each sampling site were extracted using the extraction tool in ArcGIS.

### 2.5 Statistical analysis

The data were pre-processed using Microsoft Excel 2019. The Spearman correlation analysis was conducted between environmental variables and secondary metabolites using the corrplot package in R 4.2.2. The stepwise regression was analyzed using the car package in R 4.4.2. Corresponding plots were constructed using ggplot2 package in R 4.2.2 and ArcGIS 10.7.

## 3 Results

### 3.1 Soil, climatic and geographic characteristics of sampling sites

Sampling sites were covered from the east to the west and from the north to the south of China (**Figure 2**). The longitude spanned 23.24°, and the latitude spanned 13.34°. The elevation in the study area varied from 5 to 3395 m, rising from east to west (**Table 1**).

Soil properties varied considerably between the sampling sites (**Table 1**). The average soil pH varied between 6.13 and 8.54, the average organic matter varied between 10.02 and 334.23 g kg^-1^, the average available nitrogen content ranged from 41.18 to 1,168.00 mg kg^-1^, the average available phosphorus content ranged from 5.87 to 100.23 mg kg^-1^, and the average available potassium ranged from 71.76 to 1,038.73 mg kg^-1^ (**Table 1**).

The study area spans multiple climate zones in China, including temperate, warm temperate, subtropical, and alpine. The annual mean temperature of each sampling site varied between 3.12 and 17.31°C, annual precipitation varied between 446.45 and 1,206.15 mm, and annual sunshine hours varied between 1,037.23 and 2,690.20 h (**Table 1**).

### 3.2 Purpurin and mollugin content of *R. cordifolia* roots in different locations

**Figure 3:**
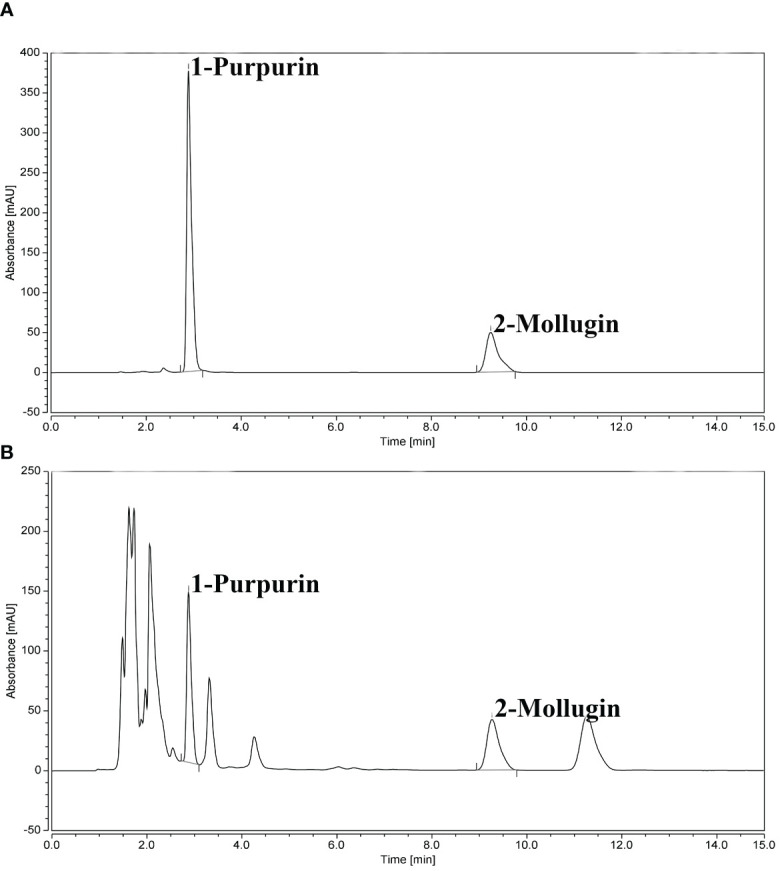
Chromatographic diagram of standard and sample solution. **(A)** Standard solution; **(B)** Sample solution.

**Figure 4:**
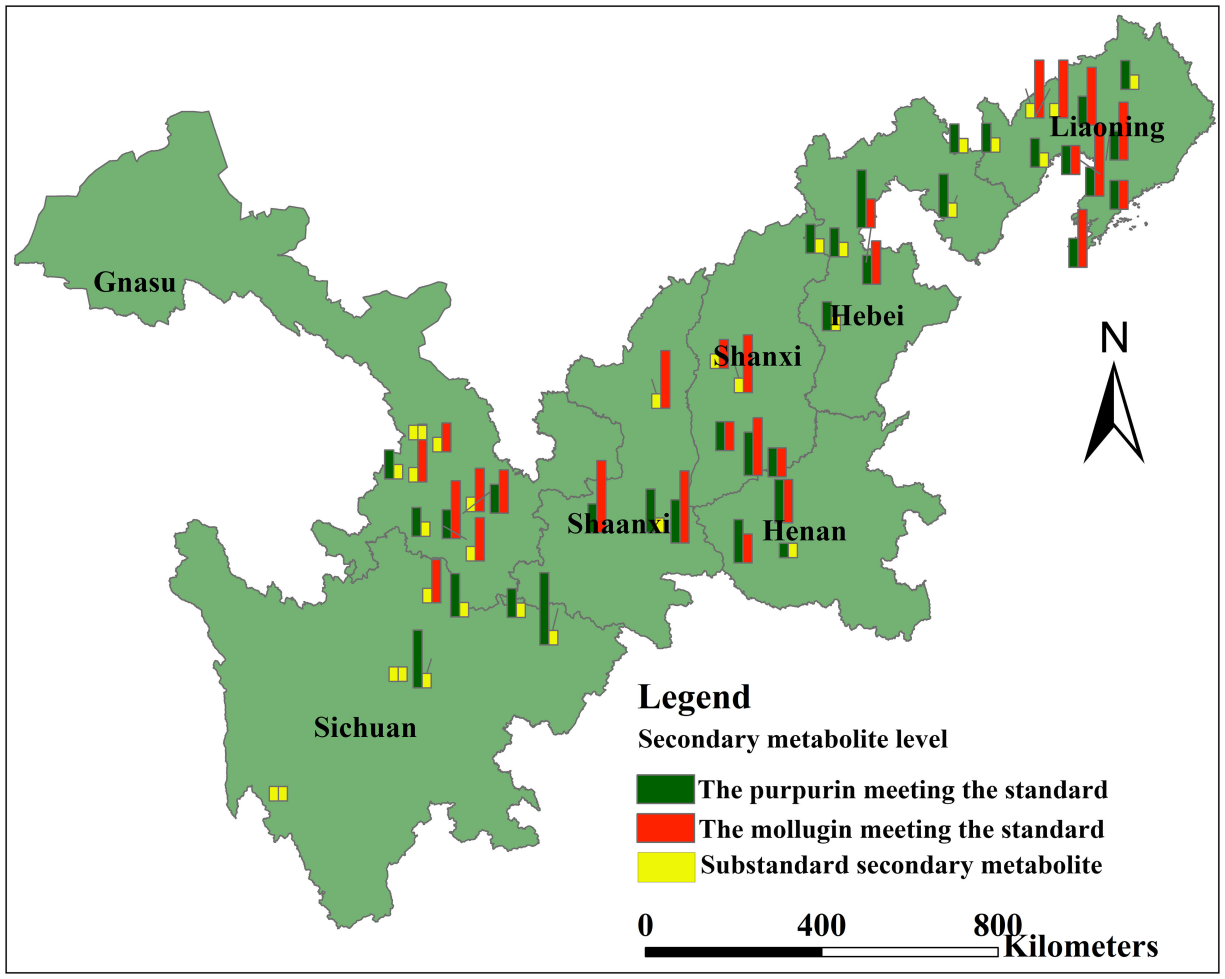
Quantitative comparison of metabolic compounds at each sampling site.

The contents of purpurin and mollugin at each sampling point were calculated according to the UHPLC chromatogram (**Table 2**). The chromatograms of standard and sample solutions are shown in **Figure 3**. The purpurin and mollugin contents in *R. cordifolia* roots varied greatly among sampling sites in the study area. Purpurin content ranged from 0.00 to 3.03 mg g^-1^, the minimum and maximum values were found at sampling points in Sichuan. The content of mollugin varied from 0.03 to 10.09 mg g^-1^. The minimum value was found in Sichuan sampling site, while the maximum value was found in Liaoning sampling site. The contents of the two secondary metabolites in *R. cordifolia* roots varied greatly in different provinces. For example, the content of purpurin in more than half of the samples in Gansu was less than 1.00 mg g^-1^, and the highest was 1.34 mg g^-1^. The content of purpurin in in the samples in Hebei was higher than 1.00 mg g^-1^, and the highest content was 2.05 mg g^-1^. However, the content of mollugin in the samples in Hebei was less than 4.00 mg g^-1^, and the highest content was 5.10 mg g^-1^. The content of purpurin in the roots of *R. cordifolia* in nearly half of the sampling sites in Sichuan was less than 1.00 mg g^-1^, and the highest was 3.03 mg g^-1^. The content of mollugin in the roots in almost all sampling sites was not up to standard, indicating that the quality of *R. cordifolia* in each sampling site in Sichuan was uneven. The content of secondary metabolites in the roots of *R. cordifolia* in Shanxi, Shaanxi and Henan provinces can basically reach the standard. In addition, the content of secondary metabolites in the roots of *R. cordifolia* in a small number of sampling points in Liaoning was not up to standard, mainly distributed in the western part of Liaoning, while the quality of *R. cordifolia* in the southeastern part of Liaoning is higher (**Figure 4**).

**Table 2:** The content of purpurin and mollugin in the roots of *R. cordifolia* at each sampling point.

Sampling site	Province	Longitude	Latitude	Purpurin content (mg g^-1^)	Mollugin content (mg g^-1^)
S1	Gansu	104.568	34.615	1.06	5.28
S2	Gansu	104.591	34.650	0.69	5.61
S3	Gansu	103.909	35.893	0.74	4.61
S4	Gansu	103.909	35.893	0.99	2.01
S5	Gansu	104.089	34.342	0.99	5.56
S6	Gansu	104.275	34.085	1.11	6.60
S7	Gansu	103.411	35.256	0.78	6.47
S8	Gansu	103.238	35.317	1.06	3.70
S9	Gansu	103.517	34.124	1.34	3.17
S10	Hebei	115.511	39.342	2.05	4.23
S11	Hebei	117.909	40.019	1.85	1.66
S12	Hebei	114.354	38.000	1.41	2.04
S13	Hebei	118.504	41.324	1.16	2.81
S14	Hebei	114.799	39.528	1.21	2.93
S15	Hebei	114.788	39.606	1.15	2.20
S16	Hebei	115.579	38.862	1.26	5.10
S17	Henan	112.601	34.112	1.65	5.67
S18	Henan	111.505	33.356	1.64	4.10
S19	Henan	112.674	33.729	0.87	3.57
S20	Henan	112.233	35.103	1.03	4.59
S21	Liaoning	122.490	40.569	1.34	6.16
S22	Liaoning	122.186	40.181	1.08	4.85
S23	Liaoning	121.972	39.873	1.50	10.09
S24	Liaoning	122.239	40.310	1.27	4.63
S25	Liaoning	123.589	41.908	1.48	2.56
S26	Liaoning	122.169	41.299	1.40	6.61
S27	Liaoning	119.391	41.221	1.05	2.93
S28	Liaoning	121.192	38.734	1.02	7.83
S29	Liaoning	120.770	41.199	1.14	2.89
S30	Liaoning	120.786	41.756	0.84	7.73
S31	Liaoning	120.786	41.756	0.60	7.59
S36	Shaanxi	109.705	36.695	0.51	6.43
S37	Shaanxi	109.979	33.865	1.68	8.45
S38	Shaanxi	107.919	34.163	1.21	8.68
S39	Shaanxi	109.374	34.114	1.80	3.04
S32	Shanxi	111.942	35.153	1.56	6.06
S33	Shanxi	111.456	37.438	0.74	4.49
S34	Shanxi	111.275	35.721	1.16	4.62
S35	Shanxi	111.881	36.896	0.77	6.03
S40	Sichuan	103.815	32.748	0.00	5.89
S41	Sichuan	104.497	32.463	1.80	0.03
S42	Sichuan	105.880	32.447	1.37	3.25
S43	Sichuan	103.616	30.989	2.87	0.37
S44	Sichuan	100.349	28.566	0.65	0.94
S45	Sichuan	106.660	31.870	3.03	0.56
S46	Sichuan	103.250	31.118	0.96	0.12

### 3.3 Correlation between purpurin, mollugin content and environmental variables

**Figure 5:**
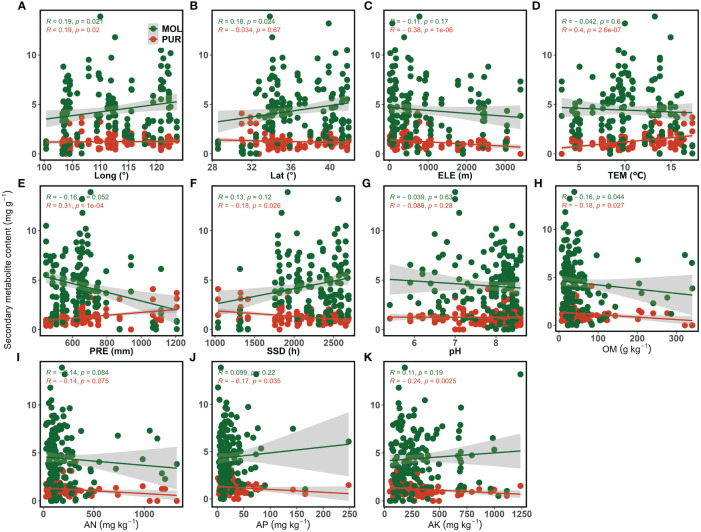
Correlation between purpurin, mollugin content, and environmental variables. **(A)** The relationship between secondary metabolite content and longitude; **(B)** The relationship between secondary metabolite content and latitude; **(C)** The relationship between secondary metabolite content and elevation; **(D)** The relationship between secondary metabolite content and annual mean temperature; **(E)** The relationship between secondary metabolite content and annual precipitation; **(F)** The relationship between secondary metabolite content and annual sunshine hours; **(G)** The relationship between secondary metabolite content and soil pH; **(H)** The relationship between secondary metabolite content and organic matter content; **(I)** The relationship between secondary metabolite content and available nitrogen content; **(J)** The relationship between secondary metabolite content and available phosphorus content; **(K)** The relationship between secondary metabolite content and available potassium content. PUR, purpurin; MOL, mollugin; Long, Longitude; Lat, Latitude; ELE, Elevation; TEM, Annual mean temperature; PRE, Annual precipitation; SSD, Annual sunshine hours; OM, Organic matter; AN, Available nitrogen; AP, Available phosphorus; AK, Available potassium.

Purpurin and mollugin contents showed different relationships with soil, climate, and topographic factors (**Figure 5**). Longitude showed a significant positive correlation with purpurin content (*p* < 0.05) (**Figure 5A**), while elevation (*p* < 0.001) showed a significant negative correlation (**Figure 5C**). Annual mean temperature (*p* < 0.001) and annual precipitation (*p* < 0.001) showed highly significant positive correlations with purpurin content (**Figures 5D, E**), whereas annual sunshine hours showed a significant negative correlation with purpurin content (*p* < 0.05) (**Figure 5F**). Organic matter content (*p* < 0.05), available phosphorus content (*p* < 0.05), and available potassium content (*p* < 0.01) were significantly negatively correlated with purpurin content (**Figures 5H, J, K**). For mollugin, longitude (*p* < 0.05) and latitude (*p* < 0.05) were significantly positively correlated with mollugin content (**Figures 5A, B**). Organic matter content (*p* < 0.05) showed a significant and negative correlation with mollugin content (*p* < 0.05) (**Figure 5H**). There was no significant correlation between mollugin content and climatic factors (**Figures 5D–F**), however, there was a negative correlation between annual precipitation and mollugin content (*p* = 0.052) (**Figure 5E**).

### 3.4 Regression of purpurin, mollugin content with environmental variables

In order to further explore the relationship between the two secondary metabolites and environmental factors, a stepwise regression model between purpurin, mollugin and environmental factors was established (**Table 3**). Model results showed that purpurin and mollugin were affected by different factors. Purpurin in *R. cordifolia* roots was affected by latitude, annual mean temperature and annual precipitation, and the three factors had a positive impact on purpurin. Otherwise, the model results showed that root mollugin content was only negatively affected by annual precipitation.

**Table 3:** Stepwise regression model between secondary metabolites and environmental factors.

Variable	Estimate	Standard Error	Z value	*P* (>|z|)	*R* ^2^
Purpurin					
Intercept	-5.821	1.114	-5.224	< 0.001	0.281
Lat	0.091	0.024	3.796	< 0.001	
TEM	0.119	0.020	5.905	< 0.001	
PRE	0.002	0.001	3.888	< 0.001	
Mollugin					
Intercept	1.258	0.296	4.245	< 0.001	0.107
PRE	-0.002	0.000	-4.386	< 0.001	

Lat, Latitude; TEM, Annual mean temperature; PRE, Annual precipitation. Data about purpurin and mollugin were transformed using case ranking method before use in the regression model.

## 4 Discussion

### 4.1 The difference of purpurin and mollugin content in *R. cordifolia* roots in different locations

*R. cordifolia* is widely distributed in China; therefore, there are several places of origin recorded in the literature, including Shanxi, Sichuan, Henan, Shaanxi, Jiangsu, Hubei, Shandong, Anhui, and Hebei (Xue et al., 2009; Yan, 2021). Several studies have investigated the medicinal components in the roots of *R. cordifolia* from different origins and found that *R. cordifolia* from Shanxi, Shaanxi, Henan, Hebei, Jiangsu, Shandong, and Anhui met the Chinese Pharmacopoeia standards (Xue et al., 2009; Yu et al., 2017). Henan and Shaanxi are recognized *R. cordifolia* producing areas (Wen et al., 2022). The purpurin and mollugin contents of *R. cordifolia* roots from eastern and central regions in this study investigated met the requirements of the Chinese Pharmacopoeia, especially in central regions. Therefore, the study confirmed that *R. cordifolia* produced in Henan, Shaanxi, and Shanxi contained higher levels of purpurin and mollugin. However, *R. cordifolia* resources in Northeastern China have not yet been investigated. In this study, *R. cordifolia* from Liaoning contained higher purpurin and mollugin contents, which met the requirements of the Chinese Pharmacopoeia, suggesting that Liaoning could be a choice for *R. cordifolia* cultivation.

### 4.2 Effects of geographic, soil, and climate factors on the content of purpurin and mollugin

Environmental factors, including soil, climate, and topography, showed different degrees of correlation with the two secondary metabolites in the roots. The effects of geographic factors on plant secondary metabolite content have been extensively reported. The content of proanthocyanidins in *Ribes nigrum* L. was positively correlated with latitude (Yang et al., 2019b), whereas harpagoside content in *S. ningpoensis* was negatively correlated with latitude (Yang et al., 2011). Different essential oil contents in *H. italicum* showed different correlations with elevation (Melito et al., 2016). In this study, purpurin and mollugin contents were significantly and positively correlated with longitude and latitude (**Figure 5**; **Table 2**), suggesting that when the environment was closer to northern and eastern China, the purpurin and mollugin contents in *R. cordifolia* roots were higher, which is similar to the findings of Weston et al. (2013). The two secondary metabolites can cause oxidative stress in cells and therefore act as natural biocides (Robertson et al., 2009; Hook et al., 2018). The higher purpurin and mollugin content found in the eastern and central regions of this study might be due to the fact that the environmental conditions in these regions cause some kind of biotic stress, which in turn leads to the synthesis of more purpurin and mollugin. Previous studies have shown that anthraquinone content in medicinal plants *Rheum officinale* Baill. (Wang et al., 2013; Yan et al., 2017; Ge et al., 2018) and *Rumex nepalensis* Spreng (Farooq et al., 2013) increased with elevation, but in the present study elevation negatively affected purpurin content in *R. cordifolia* roots, and a similar result was found in *Rheum australe* D. Don (Pandith et al., 2014). The negative correlation between secondary metabolite content and elevation may be related to factors such as ultraviolet radiation or temperature (Spitaler et al., 2006). The positive correlation between purpurin content and annual mean temperature and the negative correlation between elevation and temperature just proved this point.

Soil nutrients play a crucial role in supporting plant growth. In the case of medicinal plants, the presence of appropriate soil nutrients not only enhances their yield but also improves their overall quality. Changes in soil nutrition will inevitably lead to changes in secondary metabolites, understanding the relationship between the content of secondary metabolites and soil nutrients can guide the cultivation of medicinal plants to improve the effectiveness of medicinal plants. However, specific soil factors that cause an increase in secondary metabolite levels in medicinal plants are not the same. Soil pH may be an important factor determining the secondary metabolite content of *Echinacea purpurea* (L.) Moench (Xu et al., 2022). The available phosphorus in the soil is thought to be a key factor influencing the secondary metabolite content in *Salvia miltiorrhiza* Bunge (Liang et al., 2021). For *Rhodiola sachalinensis* A. Bor, available phosphorus and available potassium are the main factors limiting the synthesis of salidroside (Yan et al., 2004). In this study, the findings found that higher soil nutrients were not necessarily beneficial to the synthesis of purpurin and mollugin, which is similar to the study by Shen et al. (2017), where the content of anthraquinone compounds in the roots of *Rheum tanguticum* Maxim. ex Balf was higher in nutrient-poor soil. This phenomenon might be due to the fact that under nutrient-sufficient conditions *R. cordifolia* mainly carries out primary metabolism for growth, while under poor soil conditions the plants are stressed and then carry out secondary metabolism and produce secondary metabolites to alleviate the stress.

The climate is crucial for the growth and quality of medicinal plants. Studies have shown that different medicinal plants have different requirements for climatic conditions, and secondary metabolite production can be affected by climatic conditions (Liu et al., 2020). Temperature has multiple effects on the synthesis of secondary metabolites in medicinal plants. On the one hand, temperature stress may cause medicinal plants to produce secondary metabolites for defense (Dong et al., 2011); on the other hand, high or low temperatures may increase the expression of genes involved in the synthesis of secondary metabolites (Tao et al., 2017; Yang et al., 2019b). The harpagoside content in *Scrophularia ningpoensis* Hemsl (Yang et al., 2011), and the nerolidol content in *Helichrysum italicum* (Roth) G. Don (Melito et al., 2016) are correlated positively with temperature, while there is a negative correlation between high temperature and total proanthocyanidins in *R. nigrum* fruits (Yang et al., 2019b). Temperature promoted the synthesis of purpurin in the roots of *R. cordifolia*, which may be due to high temperature stress or increased expression of related genes. Precipitation also has an impact on the synthesis of secondary metabolites in plants. Typically, drought conditions stimulate the synthesis and accumulation of secondary metabolites in plants (Humbal and Pathak, 2023). However, chlorogenic acid, choline, 3,5-o-dicaffeoylquinic acid, and coumaric acid in *E. purpurea* are positively correlated with annual precipitation (Xu et al., 2022). Two secondary metabolites in the roots of *R. cordifolia* were oppositely affected by precipitation, which promoted the synthesis of purpurin but limited the synthesis of mollugin. The impact of precipitation on the secondary metabolism of medicinal plants is complex. This complexity arises due to the potential changes in soil properties, microbial fungi, and root activity that can occur as a result of precipitation (Wang et al., 2021b; Xu et al., 2022). Sunshine hours also affect the production of plant secondary metabolites. For instance, a longer duration of sunshine can increase the content of geniposidic acid in *Eucommia ulmoides* Oliv. (Dong et al., 2011) and tanshinones in *S. miltiorrhiza* (Zhang et al., 2018). However, in the present study, correlation analysis suggested that sunshine duration might have an inhibitory effect on purpurin synthesis in *R. cordifolia* root.

### 4.3 Suggestions on *R. cordifolia* planting

The cultivation of medicinal plants needs to ensure both yield and quality, so Daodi medicinal materials are recognized as high-quality Chinese medicinal products. However, as wild medicinal plants have been over-exploited, they can no longer meet market demand. Therefore, field cultivation has become the main way to ensure the market supply of medicinal plants (Kling, 2016). In the case of field planting, many factors need to be considered comprehensively, including geographical location, soil, climate, etc. First of all, it is necessary to select the appropriate planting area, which needs to consider geographical and climatic factors. Therefore, eastern and northern China can be used as a choice when planting *R. cordifolia*. Higher temperatures facilitate the synthesis of purpurin, so the temperature in the suitable area needs to be considered. Precipitation has opposite effects on the two secondary metabolites in the roots of *R. cordifolia*. When planting *R. cordifolia*, the precipitation situation needs to be weighed, and the amount of watering in the field must also be considered. In order to ensure the yield of medicinal plants, fertilization is a necessary measure. However, the synthesis of two major secondary metabolites in *R. cordifolia* roots is inhibited by soil nutrients, therefore necessary fertilization experiments need to be performed to determine the appropriate fertilization ratio.

### 4.4 Future research prospects

The two main secondary metabolites in the root of *R. cordifolia* belong to quinone compounds, among which purpurin belongs to anthraquinone compounds (Singh et al., 2021), and mollugin belongs to naphthoquinone compounds (Morita et al., 2015). The Chinese Pharmacopoeia requires that the contents of the two secondary metabolites in the roots of *R. cordifolia* should be up to standard before they can be used as medicines (Chinese Pharmacopeia Commission, 2020), so the products of *R. cordifolia* on the market are uneven. How to improve the quality when planting *R. cordifolia* has become a problem. At present, some researchers have carried out transcriptome studies on *R. cordifolia*, and found that the synthesis of anthraquinones in the roots involves the synthesis of A, B, and C rings. Among them, A and B rings are completed by the shikimic acid pathway, while the synthesis of C ring comes from isopentenyl diphosphate (Peng et al., 2018). Previous studies have confirmed that isopentenyl diphosphate is synthesized through the 2-C-methyl-D-erythritol 4-phosphate pathway in the *Rubiaceae*. Isochorismate synthase gene, o-succinylbenzoic acid gene, 1-deoxy-D-xylulose 5-phosphate reductoisomerase gene, and 1-deoxy-D-xylulose 5-phosphate synthase gene are all key genes for the synthesis of anthraquinones (Han et al., 2001). However, the synthetic pathway of naphthoquinone compounds in the roots of *R. cordifolia* has not been reported. Therefore, in the future, on the one hand, the biosynthesis pathway of anthraquinones will be explored by molecular biology methods, and key genes will be screened. On the other hand, the relationship between the key genes in the synthesis pathway of anthraquinones and naphthoquinones and the ecological environment factors should be analyzed to find the key factors, and then measures can be taken to promote the synthesis of two secondary metabolites.

## 5 Conclusions

This study found that the quality of *R. cordifolia* varied considerably depending on the region; however, in general, the quality of *R. cordifolia* from southeastern Liaoning and the border of Shanxi, Shaanxi and Henan met the Chinese Pharmacopoeia standards, whereas Liaoning proved to be a potential source of *R. cordifolia* in northeastern China. Environmental factors have different effects on the two secondary metabolites. The purpurin and mollugin contents of *R. cordifolia* roots were significantly and positively correlated with longitude and latitude, while were negatively correlated with soil nutrients. Temperature contributed to the synthesis of purpurin, but sunshine duration inhibited the synthesis. Precipitation is a key factor limiting the quality of *R. cordifolia* because it has opposite effects on the synthesis of purpurin and mollugin. To meet the requirements of the Chinese Pharmacopoeia, the limiting factors affecting the two secondary metabolites should be considered when planting *R. cordifolia* in field. In the future, it is necessary to combine the key genes in the synthesis pathway of the two secondary metabolites, further analyze the key factors affecting the synthesis of the two compounds, and then promote the synthesis of the two secondary metabolites by controlling the environmental factors to improve the quality of *R. cordifolia*.

## Data availability statement

The original contributions presented in the study are included in the article/supplementary material. Further inquiries can be directed to the corresponding author.

## Author contributions

YW: Conceptualization, Data curation, Formal analysis, Funding acquisition, Investigation, Methodology, Project administration, Resources, Software, Supervision, Validation, Visualization, Writing – original draft, Writing – review & editing. HL: Formal analysis, Resources, Writing – review & editing. SY: Data curation, Writing – review & editing. YZ: Resources, Writing – review & editing. YH: Resources, Writing – review & editing. XH: Conceptualization, Writing – review & editing. WC: Conceptualization, Investigation, Methodology, Writing – review & editing.
